# Single use Negative Pressure Wound Therapy (NPWT) system in the management of knee arthroplasty

**DOI:** 10.1186/s12891-023-06470-2

**Published:** 2023-05-05

**Authors:** Wai-Wang Chau, Kelvin Chin-Hei Lo, Lawrence Chun-Man Lau, Michael Tim-Yun Ong, Kevin Ki-Wai Ho

**Affiliations:** 1 Department of Orthopaedics and Traumatology, Chinese University of Hong Kong, Shatin, Hong Kong SAR; 2 Department of Orthopaedics and Traumatology, Alice Ho Miu Ling Nethersole Hospital, Tai Po, Hong Kong SAR; 3 Department of Orthopaedics and Traumatology, Chinese University of Hong Kong Medical Centre, Shatin, Hong Kong SAR

**Keywords:** Negative pressure wound therapy, Length of hospital stay, Skin blister formation, BMI

## Abstract

**Background:**

Wound complication, skin blister formation in particular, causes devastating consequences after total knee arthroplasty (TKA). Negative Pressure Wound Therapy (NPWT) tries to improve wound management leading to decrease length of hospital stay and better clinical outcomes. Low body mass index (BMI) could play a part in wound recovery management although lacking evidence. This study compared length of hospital stay and clinical outcomes between NPWT and Conventional groups, and factors affected and how BMI affected.

**Methods:**

This was a retrospective clinical record review of 255 (160 NPWT and 95 Conventional) patients between 2018 and 2022. Patient demographics including body mass index (BMI), surgical details (unilateral or bilateral), length of hospital stay, clinical outcomes including skin blisters occurrence, and major wound complications were investigated.

**Results:**

Mean age of patients at surgery was 69.95 (66.3% were female). Patients treated with NPWT stayed significantly longer in the hospital after joint replacement (5.18 days vs. 4.55 days; p = 0.01). Significantly fewer patients treated with NPWT found to have blisters (No blisters: 95.0% vs. 87.4%; p = 0.05). In patients with BMI < 30, percentage of patients requiring dressing change was significantly lower when treated with NPWT than conventional (0.8% vs. 33.3%).

**Conclusion:**

Percentage of blisters occurrence in patients who underwent joint replacement surgery is significantly lower using NPWT. Patients using NPWT stayed significantly longer in the hospital after surgery because significant proportion received bilateral surgery. NPWT patients with BMI < 30 were significantly less likely to change wound dressing.

## Background

Ageing population is a worldwide phenomenon meaning the demand for total knee arthroplasty (TKA) has been increasing in recent years and is expected to demand exponentially in the coming decade [1, 2]. Wound complication following TKA is not uncommon and can relay recovery, increase medical cost and reduce optimal outcome. Patient outcomes benefiting from the recent development of wound dressing in knee arthroplasty wound management are somewhat promising despite evidence to prove the efficacy is still inadequate to confirm.

Although TKA is a game changing surgery which makes the knee joints to move again, periprosthetic complications at surgical area is one of the detrimental and sometimes very difficult to treat causing devastating consequences. Hospital re-admission related to non-infectious wound complications hugely affected joint function and joint pain [3]. Surgical site management related to wound healing involves wound care and limit any wound complication, these include: (1) wound dehiscence, (2) discharging wound, (3) haematoma and (4) infection [4]. Several commonly reported complications following joint replacement are associated with wound dressing: (1) blistering of the peri-wound area (2) prolonged drainage (3) maceration (4) redness and/or irritation, and (5) infection (superficial or deep). Blistering is among the most reported complication. Blistering is caused by movement of the joint and surrounding tissue, which generates friction between the dressing and per-wound area. Blister formation is a very common wound complication associated with soft-tissue defects as one of the unfavourable clinical outcomes sometimes causes dehiscence. Blistering is also a result of collecting subcutaneous fluid and is also caused by tissue expansion from skin swelling exerting forces on the skin when changing wound dressing. Other factors affecting the wound healing progressions are, such as, patients who are obese, smoke, or have diabetes, systematic inflammatory diseases like rheumatoid arthritis [5–8].

It is common to review the wound every few days and needs to apply a new dressing whenever necessary. The introduction of “Negative Pressure Wound Therapy (NPWT)” further improves peri-operative wound management. The negative pressure dressing was first introduced by Vacuum Assisted Closure system (V.A.C. Therapy System, KCI, TX, USA (Now 3 M, St. Paul, MN, USA)) which used a sealed open-pore sponge or gauze to prepare the wound for spontaneous healing using negative pressure [9, 10]. NPWT showed successful in different medical fields such as plastic surgery, colorectal surgery and caesarean, and now in orthopaedic field especially in cases of open fractures or open wounds [11–13]. The early version of negative pressure dressing is large and bulky [2]. NPWT carries a new design which is smaller in size, less bulky, and lightweight [3].

Another factor affecting blister formation associating with NPWT is body mass index (BMI) although related discussions are still scarce. The direction is quite clear – the higher the BMI the more likely the blister formation after total joint replacement. This observation is comparatively easy to draw because the patients concerned were mostly “obese” (defined by BMI). A retrospective review on 624 patients who underwent elective TKA was carried out by our group (2020) to look for the relationship between BMI and in-hospital postoperative complications [14]. Patients with BMI ≥ 30 kg/m^2^ suffered from an increased risk of peri-prosthetic joint infection [14].

We hypothesize that (1) wound complication rate using NPWT in patients who underwent total knee arthroplasty will reach a high standard to merit its use, (2) length of hospital stay will be improved after applying NPWT compared with Conventional wound dressing, and (3) BMI is a prognostic factor in determining the efficacy of NPWT in TKA. The aims of this study are (1) review and compare the length of hospital stay, clinical outcomes in terms of blister formation, wound complications and reasons, (2) look for the factors affecting length of hospital stay in patients using either dressing protocol, and (3) explore the influence of BMI on clinical outcomes.

## Methods

### Study participants

This was a retrospective clinical record review carried out in 2 tertiary hospitals. Patients who underwent joint replacement surgery with wound dressing using negative pressure dressing protocol (Negative Pressure Wound Therapy (NPWT) group) or conventional wound dressing protocol (Conventional group) between October 2018 and June 2022 were included and reviewed through hospital electronic record system. Inclusion criteria included (1) patients who underwent total knee arthroplasty, and (2) wound dressing using either negative pressure dressing or conventional wound dressing. Exclusion criteria included (1) patients who operated other than total knee arthroplasty, and (2) used other forms of wound dressing (i.e. not continuous negative pressure or conventional). Ethical approval was obtained from the Institutional Ethics Review Committee (Ref. No.: CRE2022.302). The study protocol complied with the Declaration of Helsinki.

### Choice of dressing in patients after joint replacement

The choice of types of dressing (NPWT or Conventional dressing) used in patients after joint replacement was decided based on services provided and dressing availability. Patients who were under private care received NPWT, and patients who were under public service received NPWT where available, otherwise conventional dressing was delivered.

### Surgical techniques

**Fig. 1:**
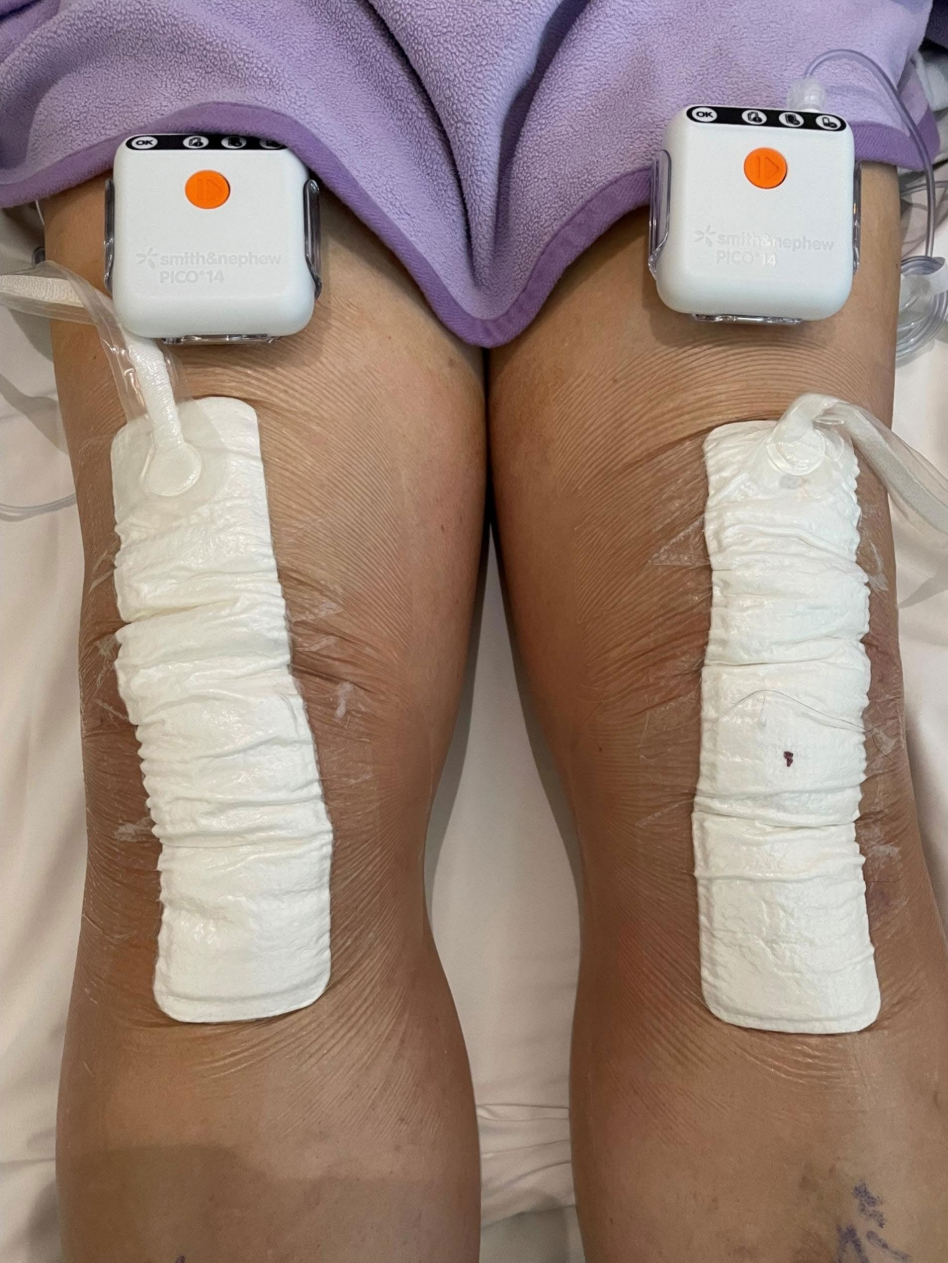
Typical application of NPWT after total knee arthroplasty

In both groups of patients, the surgical exposure, wound closure and the post-operative rehabilitation remains the same. In summary, intra-venous antibiotic was injected during induction of anaesthesia and a torniquet was applied and inflated before surgical incision. The knee was exposed through the medial para-patella approach and the patella everted during the bone cuts. After prosthesis implantation, the knee arthrotomy was closed in layers with Stratafix 1, Vicryl 2/0 and Stratafix 3/0 for skin closure. Intra-articular transamin was injected to check for leakage and haemostasis. The incision wound is further approximated with steristrips dressing before the application of either NPWT PICO™ or conventional ALLEVYN (Smith& Nephew) dressing (Fig. 1). Briefly speaking, the major difference between PICO (incisional NPWT) and conventional (traditional) NPWT is that PICO is a dressing with an aspiration system which allows the fluids to come from the wound (or suture) to the dressing (i.e. canisterless), while conventional NPWT uses a canister and the fluid goes through the foam towards the canister. An investigation comparing the biological effects between PICO and conventional NPWT concluded that PICO functioned similarly with the Conventional NPWT in terms of fluid handling, pressure transmission to the wound bed, tissue contraction, and changes in blood flow [15]. PICO delivered 80 mmHg pressure to the wound. Patients were weight bearing with walking aid and physiotherapy the next day. No restriction on the range of knee flexion were enforced. Post-operative physiotherapy rehabilitation was identical in both groups. In both groups, the dressings remained intact until follow-up clinic review at day 14 post-operation. PICO 7 was used in the earlier cases of the NPWT group, but the dressing was kept intact until follow-up clinic review at day 14 post-operation. PICO 14 was used in the majority of cases in the NPWT group. PICO 7 and 14 have no difference on indications nor contraindications. The only difference is how many days they can be used. PICO 7 has a preset of 7 days of usage and the negative pressure machine will automatically stop on day 7. PICO 14 has a preset of 14 days of usage and the negative pressure machine will automatically stop on day 14.

All patients were operated or supervised by the senior surgeon That ensured the standard of surgical procedure and patient care were consistent in all our patients regardless of the type of dressing used. As a result, the only difference between the NPWT group and Conventional group was the choice of wound dressing.

### Data collected

Medical records and perioperative parameters of eligible patients within the study period were reviewed. Patient demographics (age at surgery, sex, body mass index (BMI)), primary medical history, operated side were recorded. Surgical details including types of operation and unilateral or bilateral were collected. Length of hospital stay (days) in all patients and by laterality of operation were described. Clinical outcomes concerned about blisters (Yes or No), main wound complications, and dressing change and reasons. Blisters defined as the collection of fluid under the epidermis within 10 cm of the incision [16].

### Data collection time point

Patient demographics, primary medical history, operated side, surgical details and length of hospital stay were collected at peri-operative stage and at discharge referencing to electronic medical records in the hospital. Clinical outcomes and wound status were examined after 6 weeks of joint replacement.

### Statistical analysis

All statistical analyses were carried out by comparing NPWT group and Conventional group, unless indicated. In baseline demographics, age at surgery, sex (male/female), BMI, primary medical history (confirmed diabetic or not diabetic) and side (Left/Right) were compared between the two groups using Student T-test (numeric data) or Chi-square test (categorical data) where appropriate. Surgical details and clinical outcomes were also compared between these two groups. Days of hospital stay in patients treated with NPWT or conventional dressing who underwent either unilateral or bilateral joint replacement were compared using Student’s T-test. Furthermore, All statistical analysis will be carried out using IBM SPSS version 28 (Armonk, NY: IBM Corp). A two-sided p-value ≤ 0.05 was considered statistically significant.

## Results

A total of 255 patients were included in this study, of which 160 were treated with NPWT and 95 were treated with conventional dressing. In patients who were treated with NPWT, 96 (60.0%) underwent total knee arthroplasty (TKA) followed by 5 (3.1%) unicompartmental knee arthroplasty (UKA). Fifty-eight (36.3%) patients underwent bilateral joint replacement. One (0.6%) patient required revision. In patients who were treated with conventional dressing, 68 (71.6%) underwent total knee arthroplasty (TKA) followed by 5 (5.3%) unicompartmental knee arthroplasty (UKA). Twenty (21.1%) patients underwent bilateral joint replacement. One (1.1%) patient required revision.

### Baseline demographics

Mean age of patients at surgery was 69.95, of which 66.3% (N = 169) were female. Mean BMI was 27.25. Thirty-six (14.1%) patients were diabetic. No statistical difference in age, sex, BMI and side of operation between the 2 groups (Table 1). Significantly more patients receiving conventional dressing were diabetic (61.1% vs. 38.9%, p < 0.01).

**Table 1:** Baseline demographics of the 255 patients

	NPWT (N = 160)	Conventional method (N = 95)	P value
Age at surgery	69.94 ± 8.33	69.96 ± 6.44	0.99
Sex			
Male	60 (69.8) (37.5)	26 (30.2) (27.4)	0.10
Female	100 (59.2) (62.5)	69 (40.8) (72.6)	
BMI	27.07 ± 4.38	27.77 ± 4.12	0.31
<30	128 (76.6) (84.8)	39 (23.4) (73.6)	0.10
≥30	23 (62.2) (15.2)	14 (37.8) (26.4)	
Unknown	9	42	
Primary medical history			
Diabetic	14 (38.9) (8.8)	22 (61.1) (23.2)	< 0.01*
No	146 (66.7) (91.3)	73 (33.3) (76.8)	
Side			
Left	83 (62.4) (51.9)	50 (37.6) (52.6)	1.00
Right	77 (63.1) (48.1)	45 (36.9) (47.4)	

NPWT: Negative pressure wound therapy

BMI: Body mass index

### Surgical details

Within the 174 patients who underwent unilateral surgery, 164 (94.3%) patients received total knee replacement and the rest received unicompartmental knee replacement (Table 2). Seventy-eight (30.6%) out of all patients (N = 255) received bilateral replacement. Two patients required revision. Patients underwent bilateral joint replacements were more likely to be treated with NPWT (36.3% vs. 21.1%) and patients who underwent unilateral joint replacement were more likely to be treated with conventional dressing (78.9% vs. 63.7%). The comparison showed statistical significance (p = 0.02).

**Table 2:** Surgical details

Operation	NPWT (N = 160)	Conventional method (N = 95)	P value
UKA	5 (50.0) (3.1)	5 (50.0) (5.3)	0.08
TKA	96 (58.5) (60.0)	68 (41.5) (71.6)	
Bilateral	58 (74.4) (36.3)	20 (25.6) (21.1)	
Revision	1 (50.0) (0.6)	1 (50.0) (1.1)	
UKA + PFA	0	1 (100.0) (1.1)	
Unilateral	102 (57.6) (63.7)	75 (42.4) (78.9)	0.02*
Bilateral	58 (74.4) (36.3)	20 (25.6) (21.1)	
Length of hospital stay (days)			
All patients	5.18 ± 0.87	4.55 ± 2.11	0.01*
Unilateral	4.84 ± 0.70	4.50 ± 2.16	0.19
Bilateral	5.79 ± 0.83	4.78 ± 1.93	0.04*

UKA: Unicompartmental knee arthroplasty

TKA: Total hip arthroplasty

PFA: Patellofemoral arthroplasty

Patients treated with NPWT stayed significantly longer in the hospital after joint replacement (5.18 days vs. 4.55 days; p = 0.01) (Table 3). The statistical significance was mainly contributed by patients who underwent bilateral joint replacement (NPWT group vs. Conventional group = 5.79 days vs. 4.78 days; p = 0.04).

**Table 3:** Comparing length of hospital stay (days) between patients who underwent unilateral and bilateral knee surgeries by patient groups

Patient group	Unilateral	Bilateral	P value
NPWT	4.84 ± 0.70	5.79 ± 0.83	< 0.01*
Conventional method	4.50 ± 2.16	4.78 ± 1.93	0.62

NPWT: Negative pressure wound therapy

### Clinical outcomes (Table 4)

Significantly fewer patients treated with NPWT found to have blisters (No blisters: 95.0% vs. 87.4%; p = 0.05). No main wound complication was found in NPWT group, while 6.3% felt itchy at the wound, 6.3% of the would were soiled, and 3.3% felt pain, rash, or soaked (p < 0.01). Four (2.5%) patients with NPWT required dressing changes, or which 2 found to have air leak, 1 with blisters and 1 with machine wet. Twenty-three (24.2%) patients with NPWT change, of which 9 needed to review their wounds, 7 wounds were contaminated, itchiness, or loosened, 3 wounds with blisters and 4 wounds with other reasons.

**Table 4:** Clinical outcomes

	NPWT (N = 160)	Conventional method (N = 95)	P value
Blisters			
Yes	8 (40.0) (5.0)	12 (60.0) (12.6)	0.05*
No	152 (64.7) (95.0)	83 (35.3) (87.4)	
Main wound complications			
None	160 (69.6) (100.0)	70 (30.4) (73.7)	< 0.01*
Itchiness	0	6 (100.0) (6.3)	
Soiled	0	6 (100.0) (6.3)	
Wound review	0	10 (100.0) (10.5)	
Pain, rash, soaked or re-admitted	0	3 (100.0) (3.3)	
Dressing change and reasons			
Yes	4 (14.8) (2.5)	23 (85.2) (24.2)	< 0.01*
Reasons			
Air leak	2 (100.0) (1.3)	0	< 0.01*
Blisters	1 (25.0) (0.6)	3 (75.0) (3.2)	
Machine wet	1 (100.0) (0.6)	0	
Wound review	0	9 (100.0) (9.5)	
Contaminated, itchiness, loosened	0	7 (100.0) (7.4)	
Change after shower, others	0	4 (100.0) (4.2)	
No	156 (68.4) (97.5)	72 (31.6) (75.8)	

NPWT: Negative pressure wound therapy

No patients with blisters were treated with antibiotics and all heals without additional surgical intervention. Fluid impregnation of the dressing was not found in any patient.

### Effect of BMI on clinical outcomes (Table 5)

In patients with BMI < 30, percentages of patients not having any sign of main wound complications were significantly higher in NPWT group than Conventional group (82.1% vs. 17.9%, p < 0.01). The result was in contrary to patients with BMI ≥ 30 (69.7% vs. 30.3%, p = 0.06). Only one patient with BMI < 30 treated with NPWT required dressing change because of getting wet in the vacuum machine, while 3 patients treated by conventional dressing required dressing change, of which 2 were caused by air leakage and 1 with wound wetting. The difference in within-group percentages of dressing change for patients with BMI < 30 statistical significance (dressing change = Yes; NPWT vs. Conventional = 0.8% vs. 33.3%; p < 0.01). No statistical significance was found in BMI ≥ 30.

**Table 5:** Clinical outcomes by BMI cut-off at 30

Clinical outcomes	BMI					
	< 30		P value	≥ 30		P value
	NPWT (N = 128)	Conventional method (N = 39)		NPWT (N = 23)	Conventional method (N = 14)	
Blisters						
Yes	6 (60.0) (4.7)	4 (40.0) (10.3)	0.25	2 (50.0) (8.7)	2 (50.0) (14.3)	0.63
No	122 (77.7) (95.3)	35 (22.3) (89.7)		21 (63.6) (91.3)	12 (36.4) (85.7)	
Main wound complications	N = 128	N = 39		N = 23	N = 14	
None	128 (82.1) (100.0)	28 (17.9) (71.8)	< 0.01*	23 (69.7) (100.0)	10 (30.3) (71.4)	0.06
Itchiness	0	3 (100.0) (7.7)		0	0	
Soiled	0	4 (100.0) (10.3)		0	2 (100.0) (14.3)	
Wound review	0	4 (100.0) (10.3)		0	0	
Pain, rash, soaked or re-admitted	0	0		0	2 (100.0) (14.3)	
Dressing change and reasons	N = 128	N = 39		N = 23	N = 14	
Yes	1 (7.1) (0.8)	13 (92.9) (33.3)	< 0.01*	3 (75.0) (13.0)	1 (25.0) (7.1)	1.00
No	127 (83.0) (99.2)	26 (17.0) (66.7)		20 (60.6) (87.0)	13 (39.4) (92.9)	
Reasons	N = 1	N = 13		N = 3	N = 1	
Air leak	0	0	< 0.01*	2 (100.0) (8.7)	0	0.32
Blisters	0	1 (100.0) (7.7)		0	0	
Machine wet	1 (100.0) (0.8)	0		1 (100.0) (4.3)	0	
Wound review	0	0		0	0	
Contaminated, itchiness, loosened	0	5 (100.0) (38.5)		0	0	
Change after shower, others	0	7 (100.0) (53.8)		0	1 (100.0) (7.1)	
No	127 (83.0) (99.2)	26 (17.0) (66.7)		20 (60.6) (87.0)	13 (39.4) (92.9)	

## Discussion

Joint replacement is effective in treating end stage knee and hip osteoarthritis. One of the most detrimental comorbidities after the surgery, wound complication, hugely dictates the clinical outcomes. Although the percentage of wound infection is comparatively low, serious wound infection needs extra clinical interventions and that could result in unsatisfactory outcomes in terms of delayed wound healing, unfavourable scar appearance and thickness, and cosmetic appearance. Moreover, formation of blister happens when epidermis is separated from the skin dermis and results from continued to exert friction on the skin [17]. Wound dressing are usually applied for a relatively long period of time over the operated joint. Joint movement causes friction between the skin and dressing surface causing a shear force. Other possible factors on blister formation include (1) skin changes in older patients, (2) soft tissue oedema formation following surgery, (3) type of dressing used, and (4) mode of application of the dressing [17]. In conventional method, cotton gauze fabric and foam dressing are widely used in wound care because of their absorption power and cost-effectiveness. These products create a moist environment and protect the wound from bacteria infection. These products also adsorb the excessive exudate and remove oedema. In contrast, NPWT delivers a continuous negative pressure to the suture site/wound area. Mechanisms of NPWT include (1) promote macrodeformation by drawing wound edges together and microdeformation by facilitating cell proliferation, (2) removes excessive exudate and maintain a moist-balanced environment to promote wound healing, and (3) improves perfusion and reduces gap between capillaries and cells thus increasing blood flow to the suture site area. Result from an in-vitro study showed that lateral tension on closed incisions has also been reduced [18].

We introduce NPWT in our surgical wound site protocol aiming at further improving peri-operative wound management. This is a comparative study on the outcomes of joint replacement patients using either NPWT or conventional dressing after surgery. This study recruited a total of 255 patients, of which 160 were treated with NPWT and 95 were treated with conventional dressing. Baseline demographics, surgical details, and clinical outcomes were compared between NPWT group and Conventional method group. Next, length of hospital stay (days) between patients who underwent unilateral and bilateral knee surgeries by patient groups were compared. Furthermore, all patients were stratified by BMI cut-off at 30. Clinical outcomes between NPWT group and Conventional group were further compared in patients with BMI < 30 and in patients with BMI ≥ 30. Percentage of occurrence of blisters were significantly lower after using continuous negative pressure dressing. No main wound complication was found in this group of patients. Formation of blisters were not linked to fluid impregnation of the dressing. Patients using negative pressure dressing stayed significantly longer in the hospital after surgery because significantly higher number of them received bilateral knee surgery. In patients of BMI < 30, patients treated with NPWT were less likely to induce wound complication and change wound dressing than patients treated with conventional dressing.

Results on comparing skin blister occurrence in surgical wound management between NPWT and conventional wound therapy are still debatable. A review from Kim’s team published in year 2019 comparing the efficacy between NPWT and conventional wound management in patients who underwent TKA showed the rate of skin blisters was higher in NPWT (odd ratio: 4.44) [13]. Further looking into the results showed that comparing the weighted mean differences of skin blister subgroup analyses between NPWT and conventional wound dressing showed statistical insignificant results when performing TKA (Odds ratio: 5.39 (95% confidence interval: 0.97–29.90); p = 0.05) and the p value for the pooled comparison was 0.32. These observations showed that there was no difference in blister formation occurrence between NPWT and Conventional wound dressing despite interestingly one of the major conclusions from this study was that skin blisters were more likely to develop in patients who applied NPWT [19]. An observational study (2020) on comparing the wound outcomes of 97 patients who applied NPWT after undergoing TKA were compared with 199 patients who underwent the same surgical procedure but used conventional dressings showed that there was no statistical difference on the percentages of blister formation between NPWT (1.0%) and Conventional dressing (4.5%) groups (p = 0.17) [20]. Later, a Cochrane review on 18 clinical trials on the use of NPWT for surgical wounds healing by primary closure summarized that the formation of blisters after using NPWT was “uncertain” in the first report published in year 2020 to “might increase” the number of people with skin blistering [21] after surgery in an updated report [22]. The authors, after the updated report published in year 2022, still admitted that the evidence on this conclusion was at low-certainty and studies evaluated NPWT were from a wide range of surgeries, including orthopaedic, obstetric, vascular and general procedures [22]. A case of a 72-year-old man who presented with end stage knee OA formed a massive haemorrhagic blister sac following TKA alerted us on how important wound management is for patients coming through joint replacement surgery [23]. In a matched cohort study (2021) comparing surgical site complications with NPWT with conventional wound dressing, the mean BMI of patients received NPWT was 40.0 [24]. We further investigate the reasons for this conclusion by reviewing results from earlier studies. In an early prospective randomized trial (year 2011) in 51 patients comparing the number of days to a dry wound after joint replacement using negative pressure dressing compared with sterile gauze dressing, this study was prematured due to statistical significance found in much higher percentage of blister occurrence in patients covered with NPWT (NPWT: 63% (15/24 knees) vs. Sterile gauze application: 12% (3/36 knees)) [25]. The friction at the transition point from foam to adhesive tape was the reason suggested by the authors. We should be aware that this study aimed at recruiting obese patients (BMI ≥ 30) and patients with deep vein thrombosis on enoxaparin sodium [25]. A pilot randomized controlled trial on comparing the potential outcomes on 87 obese women undergoing elective caesarean section receiving NPWT with standard (conventional) dressing was published in year 2014 [26]. Percentages of obese women receiving NPWT was 9.1% and 2.3% in patients receiving standard dressing. The relative risk was 3.91 (0.46–33.58) and the comparison did not show statistically significant (p = 0.21) [26]. Years after, a European single centre, randomised controlled, single-blind trial on 110 suspected diagnosis of aseptic loosening of prosthesis (56 patients with hip prosthesis aseptic loosening and 54 patients with knee prosthesis aseptic loosening) was reported in year 2018 to compare the effectiveness in wound healing of patients using NPWT versus conventional dressing in patients who underwent joint (hip or knee) replacement [27]. 88% of patients receiving NPWT found no blister and 70% of patients receiving conventional dressing did not have skin blister and statistical significance was reached (p < 0.05) [27]. Mean BMI in NPWT group was 27.7 and Conventional group was 28.2, which indicated the patients were overweight to obese. In a recent article published in year 2019, a total of 160 (80 closed incisional NPWT and 80 control (silver-impregnated occlusive dressing)) consecutive patients who were scheduled to undergo revision joint replacement were elected to enter a prospective, randomized, controlled trial [28]. No blister occurrence was found in NPWT group and 1 found in control group and the comparison did not show statistical significance. One of the selection criteria in this study was that body mass index was greater than 35, and results showed mean BMI was 31.9 in NPWT group and 33.4 in Control group [28]. Interestingly, the subjects selected in these aforementioned studies were targeted to be overweight to obese. The results were conflicting between earlier studies and recent studies. A case series published in year 2021 showed significantly lower percentage of blister formation in patients treated with modified negative pressure wound therapy than conventional therapy (3.7% vs. 27.3%, p = 0.04) [29]. In our study, after stratifying the clinical outcome by BMI cut-off at 30, no statistical significance was found in percentage of blister occurrence between NPWT group and Conventional group for patients with BMI < 30 or BMI ≥ 30, which indicates no statistical difference in blister formations regardless of BMI. Majority of earlier studies on comparing NPWT with conventional dressing method mainly concerned about overweight to obese patients, usually defined as patients with BMI ≥ 30. Before controlling for BMI, blister formation was significantly lower when using NPWT on wound management (Table 4). No wound complication was found and there was significantly lower percentage of dressing change. After controlling for BMI, the advantages of significantly lower wound complication rate and dressing change rate remain in patients with BMI < 30. We speculate that using NPWT has definite advantages on decreasing the risk of blisters, incidence of wound complication and likelihood of changing dressing in patients with BMI lower than 30. Further cohort studies and randomized trials on similar studies recruiting patients with comparatively lower BMI values were warranted. In summary, the association between NPWT and blister formation was still a debatable topic and that needs to be further explored. The chance of blister formation in patients with BMI < 30 kg/m^2^ also needs to be explored.

Another important finding in this study is that our patients using negative pressure dressing stayed significantly longer in the hospital after surgery. Bilateral joint replacement was performed in 30.6% (78 out of 255 patients) of our patients, of which 74.4% received negative pressure dressing and 25.6% used conventional dressing. In our patients who underwent bilateral joint replacement, the mean number of days of hospital stay was 5.79 days for those received NPWT and 4.78 days in patients treated with conventional dressing and the comparison showed statistically significant (p < 0.01). Length of hospital stay of patients who underwent TKA or orthopaedic surgeries treated with NPWT has been discussed for years. Studies found that the number of days has been significantly decreased after using NPWT [19, 20, 30, 31]. A meta-analysis on NPWT for closed incisions in orthopedic surgeries, however, concluded that there was no statistically significant difference in the length of hospital stay when using NPWT in orthopaedic trauma surgeries [32]. An article published in year 2017 from a regional total joint replacement centre summarized that patients who underwent bilateral joint replacement required to stay in the hospital for 8.1 days and 6.7 days in patients who underwent unilateral operations [33]. Of all patients, 25.4% of patients were with BMI ≥ 30 and no statistical difference on length of hospital stay was found between patients with BMI ≥ 30 and < 30 [33]. This conclusion was based on a comparison among the selected 3 studies [34–36] and the authors admitted that the generalizability of this conclusion was limited by small sample size [32]. These findings coincide with our findings which can conclude that the longer days of hospital stays in our bilateral patients is a natural finding. Our significantly more hospitalization days in our patients treated with negative pressure dressing is a coincident event not a cause of dressing type. Using continuous negative pressure dressing is found to be significantly better than conventional dressing in terms of multiple clinical outcomes. The benefits from using continuous negative pressure dressing outweighs the longer length of hospital stay after using it. To our knowledge, this is the first study to report the relationship among the use of NPWT, laterality of the joint replacement surgery and length of hospital stay. Further study looking into the influence of baseline characteristics and prognostic factors on length of hospital stay in BMI < 30 patients who underwent total knee arthroplasty (TKA) or unicompartmental knee arthroplasty (UKA), unilateral or bilateral and different dressing types is recommended. Further investigation on the efficacy of applying NPWT on reducing the length of hospital stay and the factors affecting the number of days is worth sorted.

### Limitations of this study

The present study incurs the disadvantages of retrospective study. Missing data leads to differences in the number of subjects in our further comparisons, for example, stratification by BMI cut-off value. This is because we could not find details on body height and body weight in some patient records, despite the effect was not detrimental to conclude the results. Small sample size, selection bias and confounding might have been accounted for in this study. The advantage of using NPWT on decreasing blisters incidence, wound complication and changing dressing in patients with BMI lower than 30 might have been a result of chance. We have confidence to support this finding because the confounding factor, BMI, has been controlled, although the possibility cannot be totally ruled out. In view of this, further studies have been suggested to further prove the observations.

## Conclusions

This study shows that the percentage of blisters occurrence in patients who underwent joint replacement surgery is significantly lower after using NPWT. No main wound complication was found in these patients. Patients using NPWT stayed significantly longer in the hospital after surgery because significantly higher number of patients received bilateral knee surgery. Further studies on recruiting patients with comparatively lower BMI values (BMI < 30) through a cohort study or a randomized controlled trial are recommended.

## Data Availability

The datasets used and/or analysed during the current study are available from the corresponding author upon request.

## List of Abbreviation

TKA: Total Knee Arthroplasty
KL: Kellgren and Lawrence Grading Scale
NPWT: Negative Pressure Wound Therapy
OA: Osteoarthritis
BMI: Body mass index
